# Analysis of Biomolecules Based on the Surface Enhanced Raman Spectroscopy

**DOI:** 10.3390/nano8090730

**Published:** 2018-09-15

**Authors:** Min Jia, Shenmiao Li, Liguo Zang, Xiaonan Lu, Hongyan Zhang

**Affiliations:** 1 Shandong Provincial Key Laboratory of Animal Resistance Biology, Institute of Biomedical Sciences, Key Laboratory of Food Nutrition and Safety of Shandong Normal University, College of Life Science, Shandong Normal University, Jinan 250014, China; mjia@sdnu.edu.cn (M.J.); zangliguo@163.com (L.Z.); 2 Beijing Advanced Innovation Center for Food Nutrition and Human Health, Beijing Technology and Business University (BTBU), Beijing 100048, China; 3 Food, Nutrition and Health Program, Faculty of Land and Food Systems, The University of British Columbia, Vancouver, BC V6T 1Z4, Canada; shenmiao.ivy.li@gmail.com (S.L.); xiaonan.lu@ubc.ca (X.L.)

**Keywords:** biomolecule, surface enhanced Raman spectroscopy (SERS), nanomaterial, analysis, identification, biology

## Abstract

Analyzing biomolecules is essential for disease diagnostics, food safety inspection, environmental monitoring and pharmaceutical development. Surface-enhanced Raman spectroscopy (SERS) is a powerful tool for detecting biomolecules due to its high sensitivity, rapidness and specificity in identifying molecular structures. This review focuses on the SERS analysis of biomolecules originated from humans, animals, plants and microorganisms, combined with nanomaterials as SERS substrates and nanotags. Recent advances in SERS detection of target molecules were summarized with different detection strategies including label-free and label-mediated types. This comprehensive and critical summary of SERS analysis of biomolecules might help researchers from different scientific backgrounds spark new ideas and proposals.

## 1. Introduction

**Figure 1:**
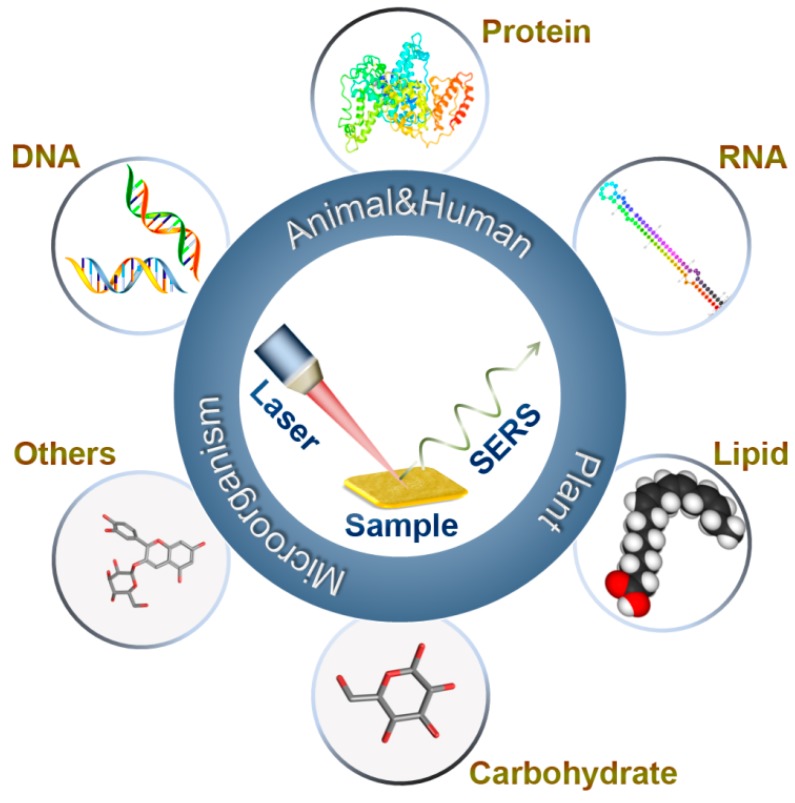
SERS detection of biomolecules.

The analysis of biomolecule is significant in various application fields, such as clinical diagnostics, food safety analysis, environmental monitoring and pharmaceutical development [1,2,3,4]. Biomolecules are the substances present in living organisms that play important roles in chemical and biological processes, including macromolecules (e.g., proteins, nucleic acids, carbohydrates, lipids) and small molecules (e.g., primary metabolites, natural products) [5,6,7]. Many detection methods have been employed to determine the biomolecules in vitro or in vivo, such as chromatography-mass spectroscopy [8], enzyme-linked immunosorbent assay (ELISA) [9], colorimetric and fluorescence detection [10,11], polymerase chain reaction (PCR) [12,13], and vibrational spectroscopy (e.g., infrared and Raman spectroscopy) [14]. This review focuses on the latest advances of surface enhanced Raman spectroscopy (SERS) in the analysis of biomolecules in humans, animals, plants and microorganisms over the past ten years (Figure 1).

SERS provides complementary analysis on molecular identification or quantification because it can reveal the information of complete structure or accurate amount [15]. Nanomaterials have been introduced into SERS methods as substrates and nanotags to improve the sensitivity and selectivity of the detection with excellent enhancement factor that can realize single target molecule detection [16]. With the development of nanotechnology, increasing varieties of nanomaterials were discovered, including noble metal (e.g., Au and Ag) and transition metal (e.g., Co and Pt) with different material morphologies, such as nanoparticles (NPs), nanoflowers, nanoclusters, and nanostars. Due to the synergistic effects, many nanohybrids were designed with non-metallic material, such as SiO_2_ [17] and graphene oxide (GO) [18]. Numerous complex structures were designed to enhance the SERS signal, containing hexagonal-packed lotus seedpod like array substrate [19], Fe_3_O_4_-Au core-shell NPs with branched gold shell [20] and Si nanopillars (SiNPLs)@silver nanoparticles (AgNPs) [21], etc.

Based on the structures of analyte molecules, different SERS detection strategies, including label-free and label-mediated types, were developed to achieve sensitive and accurate analysis. Label-free strategies can achieve the direct detection of target molecules without any Raman labels [22]. At first, the SERS signals are directly generated due to the interaction between target molecules and SERS-active substrates. In recent years, to overcome the challenges of deciphering the complex SERS spectra and low sensitivity of the previous methods, other label-free methods, such as aggregation, were developed to realize a more sensitive detection. Analyte-induced aggregation of plasmonic NPs leaded to the formation of hot spots that can enhanced the Raman scattering signals because of the particle size increase [23]. Due to the difficulty in direct detection of biomolecules, label-mediated detections were applied to specific detection by the use of antibodies or aptamers [24,25]. The general label-mediated strategies were immunoassay-based strategies adopting antigen-antibody reaction and SERS nanotags in SERS analysis to improve the sensitivity and selectivity of target detection. SERS nanotags were prepared by the combination of Raman reporter, metallic NPs and target recognition elements. Besides the general antibodies, aptamers were also used as the recognition elements. With the high specific biorecognition of aptamer and its unique DNA feather such as the rolling circle amplification technique, aptamer-based strategy for constructing SERS biosensors has attracted increasing attention in biomolecule detection.

In this review, we highlighted the application of SERS detection of biomolecules that are body components of human, animal, plant and microorganism. The SERS detection methods were summarized and classified based on the natural property of target molecules.

## 2. Application on the Detection of Human and Animal Original Biomolecule

### 2.1. Protein

The accurate and sensitive detection of proteins is crucial for proteomics and therapeutic research. Proteins are essential parts of organisms that are involved in virtual process with various functions. Therefore, structure analysis and quantitation of major species of functional proteins are critically important for understanding their functions. Meanwhile, there is an urgent demand for rapid and sensitive analysis of disease biomarkers for the early diagnosis of diseases and raising the survival rate of patients.

#### 2.1.1. Crucial Functional Proteins

Albumins generally act as transport proteins for numerous compounds in blood plasma [26]. Bovine serum albumin (BSA) was chosen as a model molecule to research the enhancement of SERS-active substrates in label-free detection. Noble metallic nanomaterials such as gold nanocylinders [22] and SiO_2_/Au nanoshells [17] were used as substrates for BSA detection. To keep proteins in their natural structure and conformation, in-liquid SERS detection of protein was based on nanoparticle aggregating as SERS-active hot spots for label-free detection [27]. Foti et al. [26] applied optically induced gold nanorods aggregation to the detection of BSA in liquid by combining light scattering, plasmon resonance and SERS. Other Raman resonant biomolecules such as catalase and hemoglobin were used to investigate the application of this methodology, and the limits of detection of catalase and hemoglobin were much lower than that of BSA.

Hemoglobins are a kind of iron-containing metalloproteins that transport oxygen in erythrocytes of all vertebrates. Iron porphyrins in hemoglobins can be an intrinsic selective probing which shows resonance enhanced Raman spectrum when excited with a laser in Soret and Q-band regions [15]. Therefore, label-free detection strategy could be applied to hemoglobin detection. Silver nanomaterials, such as colloidal AgNPs [15] and nano-silver film [28], were usually used as substrates in SERS experiment to achieve excellent enhancement of Raman signal. A novel SERS nanoprobe modified with organic cyanide (4-mercaptobenzonitrile, MBN) was developed for accurate detection of oxidized hemoglobin and Fe^3+^ ions in living cell [7]. The MBN-modified, SERS-active nanopipette gave new insights into in situ single cell detection.

Enzymes, which are mostly proteins in nature and act as selective biological catalysts, were characterized by SERS. Thrombin is one kind of serine proteolytic enzyme that plays an important role in regulating blood coagulation. Due to the successful selection of the thrombin-binding aptamer with high affinity and high specificity, a lot of aptamer-based SERS methods were developed for tracing thrombin analysis with improved selectivity and sensitivity [25,29]. The thrombin-binding aptamer was used as the capture probe with selective affinity that realized the SERS application in complex matrices. Meanwhile, by using the unique catalytic activity of thrombin, the enzymatic amplification-based strategy was established to achieve sensitive and selective detection of thrombin. The addition of thrombin could catalytically cleave the multiple arginine peptides into fragments, so that the aggregates of gold nanoparticles (AuNPs) incorporating Raman reporter molecules induced by peptides was decreased [30]. As the ability of fragments to form “hot spots” being weakened, the SERS signals were sharply diminished. The limit of detection (LOD) was 160 fM, which indicated that the sensitivity of this method was improved compared with non-enzymatic amplification based methods. This strategy can also be applied to other enzymes by appropriately utilizing the catalytic properties. Kinases are a group of enzymes that catalyze phosphorylation of specific substrate proteins [31,32,33]. Using this property, the kinase was detected by monitoring the changes in the intensity of Raman peak markers before and after phosphorylation [34]. The quantification of activity level of the enzyme can be achieved utilizing the enzyme concentration based on the enzymatic amplification-based strategy [35].

Immunoglobulin, also known as the antibody, is Y-shaped protein in different varieties known as isotypes [36,37]. Immunoglobulin is generated by the immune system to counteract pathogens such as viruses [38] and bacteria [39]. Based on the inherent antigen-antibody reaction, the immunoassay-based strategy was employed to detect immunoglobulin [40]. The traditional sandwich-format assays were usually achieved by combining antibody modified SERS-active substrates to capture target immunoglobulins and extrinsic Raman labels to enhance antigen-antibody binding kinetics [41]. Neng et al. [42] utilized AuNPs coated with the antigen as the SERS-active substrate and protein A/G modified with Raman reporter molecules as a bi-functional reporter to detect the antibody of West Nile Virus. The LOD was 2 ng/mL antibodies in the serum sample. Variants of this detection platform were applied to the trace-level detection of immunoglobulin.

Cytokines are secreted proteins produced by immune system cells regulating the immune activity [43,44]. Immunoassay-based strategy was also applicable to cytokines detection. Kamińska et al. [45] developed an immunoassay-based strategy employing diatom biosilica as the SERS-active substrate and DTNB-labeled immune-AuNPs as the SERS nanotag to detect the interleukin 8 (IL-8) in blood plasma [46]. Based on this method, a SERS immunoassay combined with a microfluidic device was developed for multiplexed recognition of interleukins IL-6, IL-8, and IL-18 in blood plasma with the LOD of 4.2 pg/mL. Wang et al. [47] also developed a multiplexed immunoassay of three cytokines, interferon gamma, interleukin-2, and tumor necrosis factor alpha, based on SERS signal enhanced by the controlled assembly of “hot spot” with low LOD and large signal-to-noise ratio in complex matrix.

Hormones communicate among organs and tissues to regulate various functions as a class of signaling molecules [48]. Human chorionic gonadotropin (hCG), an important pregnancy diagnostic marker, is a glycoprotein hormone that stimulates steroid hormone and progesterone production in the luteum [49]. Liang et al. [18] developed a series of methods based on the nanogold reaction between HAuCl_4_ and H_2_O_2_ that forms AuNPs with Victoria blue 4R molecular probes to detect hCG. The concentration of hCG can influence the catalytic effect of nanozymes such as AgNPs clusters and GO. Therefore, the changes of SERS intensity were linear to the concentration of hCG. This novel strategy can be further applied to other proteins combined with the nanozyme catalysis to develop the SERS detection platform.

Receptors are protein molecules that bound with extracellular chemical signals, causing some forms of cellular/tissue responses [50]. G-protein-coupled receptor 120 (GPR120) mediated response to long chain fatty acids (LCFAs), but the mechanism of GPR120 acting in the transduction of LCFAs was uncovered. To understand the function of GPR120 in fat chemoreception, SERS-active gold nanorods conjugated with fluorescence-active CaMoO_4_:Eu^3+^ NPs and Raman reporter molecule 4-mercaptobenzoic acid were used to achieve SERS-fluorescence imaging of GPR120 in a single cell [51].

#### 2.1.2. Disease Biomarkers

Disease biomarker refers to an extracellular indicator of disease biological state or condition. These traceable substances in the organism indicate organ functions or other aspects of health. The quantitative and sensitive detection of biomarkers is very meaningful for early clinical diagnosis and evaluating the therapeutic response. Early diagnosis has great potential in improving the survival of patients with serious diseases, such as cancer, neurodegenerative disorders, and cardia-cerebrovascular diseases. SERS-based assays have attracted significant attention as a highly sensitive and non-destructive analysis with great potential in biomarker detections.

**Figure 2:**
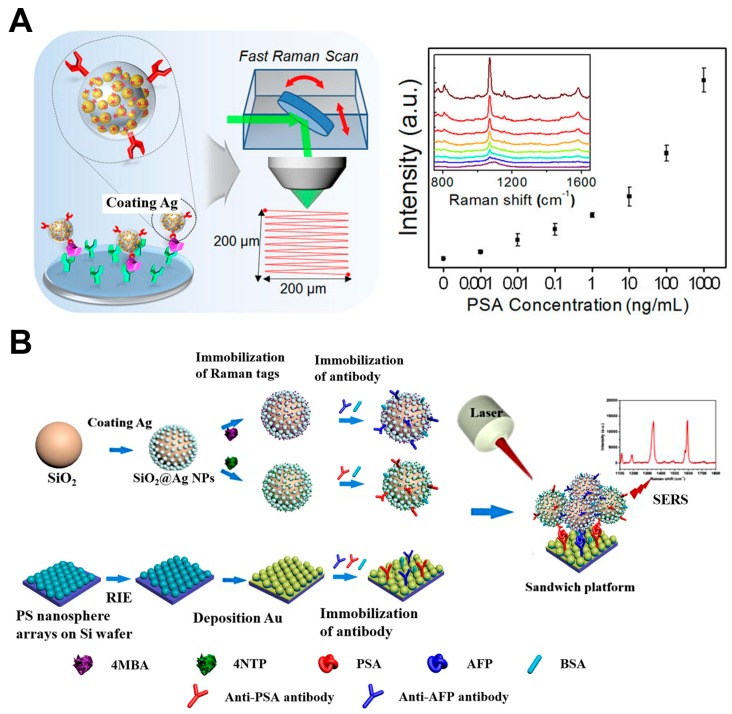
(**A**) Schematic of a SRES-based immunoassay for PSA detection [52]. (**B**) Schematic of a SERS-based multiplex immunoassay detection for PSA and AFP [16]. Reproduced with permission from [52]. Copyright American Chemical Society, 2016. Reproduced with permission from [16]. Copyright Elsevier, 2018.

Cancer is a kind of malignant disease curable only in the early stage. Cancer biomarkers, typical group of proteins, are usually over-expressed during tumor progression [16]. Therefore, tumor biomarkers have already been used for specific cancer identification, including carcinoembryonic antigen (CEA) for colorectal cancer [24], α-fetoprotein (AFP) for liver cancer [16] and prostate specific antigen (PSA) [52]. “Sandwich” structure SERS immunoassays were usually used in biomarker protein detection and involved SERS-active substrates, target proteins, and SERS nanotags [53]. The multiple SERS-active substrates were prepared by conjugating antibodies to capture target proteins [3,24,54]. Then the nanostructure-based SERS nanotags modified with Raman reporter and target proteins participated the antibody-antigen-antibody interaction. Li et al. [24] utilized gold nanobowl arrays and gold nanoshells as SERS-active substrates and SERS nanotags, respectively. This sandwich immunoassay showed a good linear relationship between CEA concentration and SERS signal intensity with a LOD of 1.73 pg/mL CEAs. PSA was detected on a glass slide with spot-arrays using SERS dot as the probe (Figure 2A). A full-area confocal raster Raman was applied to detect the SERS dot at the single probe level fixed on the glass slide by antigen-antibody interaction. This method showed high sensitivity with a LOD of 3.4 fM PSAs [52]. Besides single target detection, the SERS-based multiplex immunoassays were utilized to the ultrasensitive detection of different cancer biomarkers simultaneously. Wang et al. [16] used two kinds of Raman reporters labeled SiO_2_@Ag immune probes and gold-film hemisphere array substrate to realize multiple detection of PSA and AFP (Figure 2B). Gao et al. [55] developed a droplet-based microfluidic SERS sensor based on wash-free magnetic immunoassay technique. Using the microfluidic system integrated compartments for mixing, PSA in serum can be split and detected without any washing steps. Quantitative evaluation of PSA was realized using the Raman signals of the residual SERS nanotags in the large droplet.

C-reactive protein (CRP) has been widely studied to reveal the relevance of inflammation and cardiovascular diseases. Guo et al. [56] developed an enzymatic activation strategy combined with immunoassay analysis to activate reduction caged Raman reporters for SERS detection of CRP. Agarose beads modified with capture antibodies, CRP and horseradish peroxidase (HRP)-modified detection antibodies were used to constitute the sandwich assay. The HRP activated reduction caged LMG to generate SERS active MG for measurements. A linear curve on a logarithmic scale was obtained between CRP concentrations and SERS signals. In addition to the immunoassay strategy, label-free strategy was also suitable for CRP detection [57]. 3D AgNPs aggregates functionalized by phosphocholine were constructed to selectively capture CRP and to enhance the SERS efficiency accordingly.

Alzheimer’s disease was a chronic and progressive neurodegenerative disease causing dementia in the senior population. Amyloid-β peptide, a biomarker to diagnose Alzheimer’s disease, was hydrophobic in nature. Distinct from the above disease biomarkers, label-free strategy was usually used in SERS detection of amyloid-β. Due to synergistic effects, many nanohybrids were designed as SERS-active substrates. For amyloid-β detection, hexagonal-packed lotus seedpod like array substrate [19], biomimetic lipid membrane [58] and SiNPLs@AgNPs [21] were constructed. 3D SERS platforms [59] were also designed to enhance the “hot spot” formation in 3D. A 3D GO based SERS substrate decorated by core-shell nanoparticle was developed from hybrid 2D GO cross-linked by amine-modified PEG. The SERS enhancement factor for 3D SERS substrate was around 3.9 × 10^12^ with the LOD of 500 fg/mL amyloid-β.

Neuron-specific enolase (NSE) is a mortality predictor in traumatic brain injury patients. “Sandwich” immunoassay methods were generally used in SERS detection of NSE with different substrates. Wang et al. [60] utilized an indium tin oxide conductive glass slip with hollow gold nanospheres (HAuNPs) as the SERS substrates instead of normal silver nanomaterials. Nile blue A and NSE antibody functionalized HAuNPs were used as SERS nanotags to achieve the sensitive detection of NSE with the LOD of 0.1 ng/mL. Subsequently, lateral flow glass-hemostix (FGH) combined with Au nanocage was used as SERS substrates to detect NSE in blood plasma achieving a LOD of 0.74 ng/mL [61]. To realize rapid and low-cost clinical diagnosis, a paper-based SERS lateral flow strip was developed to detect NSE. Au nanostar@Raman Reporter@silica NPs was employed as SERS nanotag that exhibits superior performance compared to the colorimetric methods with the LOD of 0.86 ng/mL [62], indicating that paper-based SERS detection had a great potential to meet the requirement of point-of-care (POC) testing.

### 2.2. Nucleic Acid

Detection of nucleic acids such as the DNA and RNA is significant for disease diagnosis and gene therapy [63,64,65]. SERS detection was widely used in nucleic acid analysis offering simple procedures, ultrasensitive and unique structural characterization. There are two major strategies for nucleic acid detection. Label-free detection strategy based on the distinctive SERS signals from four bases of DNA/RNA. The other strategy was modifying nucleic acid with extrinsic Raman labels or constructing SERS nanotags, which have more extensive applications than label-free strategy. A series of ingenious methods based on the properties of complementarity and amplification were developed for nucleic acid detection, such as the classical “sandwich” method, the hairpin DNA-assisted method and the signal amplification method.

#### 2.2.1. DNA

**Figure 3:**
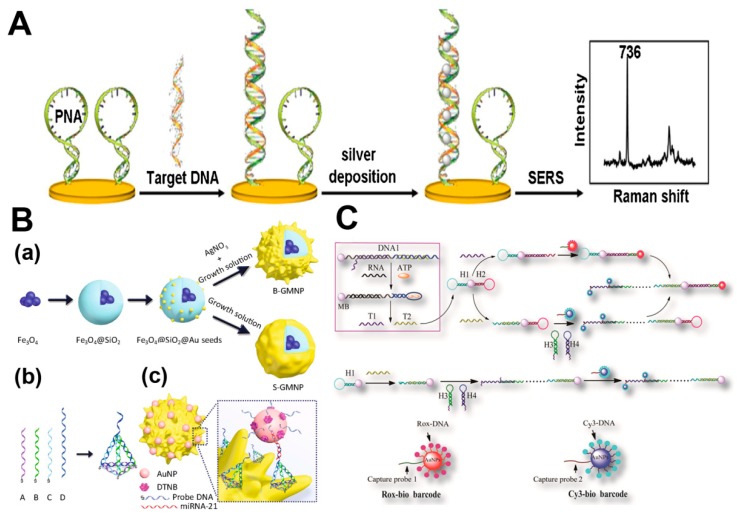
(**A**) Schematic of label-free strategy by using hairpin DNA [68]. (**B**) Sandwich strategy by using tetrahedral DNA (**a**) Schematic of B-GMNPs and S-GMNPs preparation; (**b**) tetrahedral DNA construction. (**c**) sandwich-structured strategy [20]. (**C**) Schematic of the asymmetric signal amplification SERS assay and process of HCR [77]. Reproduced with permission from [68]. Copyright Elsevier, 2018. Reproduced with permission from [20]. Copyright Springer, 2017. Reproduced with permission from [77]. Copyright American Chemical Society, 2015.

For label-free detection, the SERS signal was ordinarily generated by DNA directly absorbed on the surface of the SERS substrate. DNA bases showed specific Raman spectral which can be enhanced by the SERS-active substrate and distinctive with other impurities [66]. The facile laser scribing method was developed to fabricate AgNPs@GO composite film with a microfluidic chip for DNA detection [67]. Moreover, a unique label-free detection based on hairpin DNA and NPs in situ growth strategy was used in SERS biosensor [68]. Qian et al. utilized a peptide nucleic acid (PNA) in hairpin structure immobilized on a glass slide to recognize target DNA which can hybridize with PNA probe (Figure 3A). The duplex structure could adsorb positively charged silver ions that were chemically reduced to form AgNPs. The bases and AgNPs complex could yield a sensitive Raman signal which showed a good linear relationship with the DNA concentration. For the classical “sandwich” method, various SERS-active substrates and SERS nanotags were modified with the complementary strand to form target DNA-bridged sandwich complex. Yu et al. [69] employed two probe DNA-immobilized particles to specifically recognize the nucleotide binding sites of 683 and 735 positions on target prostate cancer antigen 3 (PCA3) mimic DNA separately via hybridization reactions. This method showed high sensitivity with the LOD of 2.7 fM PCA3. Fu et al. [70] developed a paper-based SERS lateral flow strip for sensitive detection of human immunodeficiency virus type 1 (HIV-1) DNA with Raman reporter modified AuNPs as the SERS nanotags. This method showed potential feasibility in POC self-diagnostics with LOD of 0.24 pg/mL. To improve the sensitivity of the biosensors, SERS nanotag was extensively used in DNA amplification method. Ye et al. [71] combined a triple-helix molecular switch with cascade signal amplification to achieve ultrasensitive detection of p53 gene. This amplification strategy can achieve a LOD as low as 21 aM, demonstrating a higher sensitivity.

The nucleobases are essential parts of the DNA construction and are involved in numerous processes in biology. SERS analysis was also applied to the detection of nucleobases that showed distinctive SERS signals. A microfluidic device combined with label-free SERS measurements was used to detect adenine with silver colloids as SERS substrate [72]. The prominent SERS peak at 770 cm^−1^ was caused by the ring-breathing band of adenine. The concentration of adenine showed a good linear relationship with SERS intensity quantified by the peak area of SERS peak at 770 cm^−1^. On the other hand, single nucleotide polymorphism in mitochondrial DNA (16189T→C) can also be detected by SERS analysis utilizing the ion-mediated cascade amplification strategy [73]. Target DNA binding could successfully introduce AgNPs combining with the DNA ligase reaction. By detecting the dissolved Ag^+^ from AgNPs, the LOD of targeted DNA was as low as 3.0 × 10^−5^ fm/μL of adenine.

#### 2.2.2. RNA

Similar to SERS detection of DNA, the strategies used in DNA detection were also efficient in RNA detection. For label-free detection, a variety of SERS-active substrates were designed to improve the Raman enhancement factor, such as hollow Au nanoflowers substrates [74], silver nanorod array substrates [75] and SWNTs@AgNPs [76]. Zheng et al. [76] developed an approach of DNA-templated in situ growth of AgNPs on SWNTs for the sensitive detection of a potential cancer marker miRNA-21. The silica microbeads-conjugated double stranded DNA competitively bound to the target miRNA, forming liberated ssDNA that showed high affinity to SWNTs. The liberated ssDNA-SWNT complex acted as a “nanoscaffold” for Ag^+^ to form SWNT@AgNPs upon reduction. The SWNT@AgNPs biosensor can sensitively quantify miRNA-21 with a detection limit of 5 pM. To improve the sensitivity of the SERS detection, the sandwich strategy was also employed in detecting miRNA. A novel type of Fe_3_O_4_-Au core-shell NPs with branched gold shell integrated SERS activity and superparamagnetism was designed to form the sandwich structure for the detection of miRNA-21 as shown in Figure 3B [20]. The LOD of miRNA-21 in serum was as low as 623 aM that indicated the branched nanostructures were also appropriate for SERS detection beside of smooth surfaces nanostructures. Apart from sandwich DNA nanostructures, Ye et al. [77] developed a series of signal amplification methods for miRNA-21, miRNA-141 and miRNA-203 determination. A dual functional DNA-linker-DNA probe was used for a symmetric signal amplification reaction to simultaneously analyze miRNAs.

The sensitive and simultaneous detection of multiple microRNAs can also be achieved by the application of SERS, which holds great promise for the early diagnosis of various diseases. Zhou et al. [78] utilized multiple DNA modified SERS nanotags and Ag-HMSs SERS substrate to simultaneously detect three Hepatic carcinoma related miRNAs based on the sandwich hybridization assay with a LOD of 10 fM. Shin et al. [79] developed a SERS detection combined with field-flow fractionation to sensitively detect multiple miRNAs. The target-specific polystyrene (PS) particles of three different diameters were utilized for target miRNA binding. By the polyadenylation reaction, a long tail composed of adenine was generated and the high complementariness to polythymine conjugated AuNPs led to SERS sensitivity enhancement. The three size-coded complexes were distinguished by field-flow fractionation and Raman signals obtained from the separated PS probes to measure three miRNAs simultaneously.

### 2.3. Carbohydrate

The measurement of glucose in biological systems is key to monitoring and regulating the blood glucose level for both diabetes patients and healthy individuals. To detect glucose at very low levels, the label-free strategy [80,81] and the enzyme-mediated catalytic strategy [82,83] were mainly utilized in SERS methods.

For the label-free detection of glucose, a series of SERS-active substrates were designed. Sooraj et al. [81] developed a coupled patterned Si with AgNPs arrays to improve the affinity of glucose with metal NPs. The limit of detection for glucose was low to 5 × 10^−5^ g/mL, which was much lower than the blood glucose level. For enzyme-mediated catalytic strategy, glucose oxidase was utilized to catalyze glucose producing hydrogen peroxide (H_2_O_2_) to etch the AgNPs marked with 4-mercaptopyridine, leading to the decrease of the SERS signal. The lowest detectable concentration was 10 μM [82].

### 2.4. Others

Neurotransmitters are important for information transmission in the nervous system. Catecholamines are a kind of biological amine neurotransmitters consisting of amino and catechol in structure. The sensitive and accurate detection of catecholamines is significant for brain function research and neurological diseases monitoring. Dopamine is one of catecholamine class that plays an important role in human physiology. Tang et al. [84] developed an aptamer-induced Au@Ag nanorod dimer self-assembly method to detect the dopamine ultrasensitively with a LOD of 0.006 pM. Besides the detection methods based on aptamers and antibodies, Dumont et al. [85] developed a salt-induced colloid pre-aggregation strategy to overcome the protein corona stabilization for analysis of serum samples. Moreover, the analysis of multiple catecholamines in the complex was also achieved by SERS detection. Cao et al. [86] utilized an Au-Fe Raman label for the rapid detection of dopamine, norepinephrine or epinephrine in complex serum. Moody et al. [87] developed a SERS sensor for rapid analysis of seven neurotransmitters using AuNPs and AuNPs at multiple excitation wavelengths.

## 3. Application on the Detection of Plant Original Biomolecules

Compared to the application of SERS on the detection of animal original biomolecules, there were far fewer reports about the plant originated biomolecules, which are mainly concentrated in foods or medicinal plants, such as antioxidants, anthocyanins, Chinese herbal medicinal ingredients and so on. SERS also has been applied to the molecular fingerprint identification of plant while very few applications of SERS have been explored in the basis physiological study of model plants [88,89].

### 3.1. Lipids and Antioxidant

Lipids are the main food components, which are critical for nutrition concerns [90,91]. Study on their oxidative status can help the storage and process industry. Plants are known for their antioxidative effects due to their secondary metabolites, which can be used as food additives to avoid lipid oxidation. Unsaturated fatty acids also are important for the quality and bioactivity of lipids [92,93]. For the SERS detection of lipids and antioxidants, nanomaterials such as AgNPs and AuNPs were mainly used to fabricate the SERS-active substrate using the nanofabrication methods of *e*-beam lithography [94], electroless plating [95], and so on. Interaction of targets and nanomaterials was investigated in detail to improve the sensitivity of SERS detection.

SERS have favorable potential to offer exact information of the chemical constitution of the lipid and its oxidation state. Li et al. [89] investigated the SERS assay using silver (Ag) dendrites to enhance SERS signal of detecting canola oil and alpha-tocopherol, the oxidation process of canola oil was also investigated. SERS showed better sensitivity than general Raman or ultraviolet (UV) methods in monitoring the transformation of lipid structure when oxidated. Tomato represents a major horticultural crop in human diet. Carotenoids, a kind of antioxidant rich in tomatoes, are important for plant physiology and mammalian organisms [96]. Radu et al. studied the differences of two kinds of carotenoids in tomato extracts using an *e*-beam lithography SERS-active substrate. A model sample which was a mixture of two kinds of carotenoids was processed and analyzed. Two data processing methods were used, and the result agreed with the conventional analysis methods [94].

Chan et al. [97] described the preparation of nanoarrays with AgNPs used as SERS substrates. The nanoparticle-based platform offered application prospects in SERS analysis of beta-carotene, and the detection limit was <0.63 ppm. Hsueh et al. [95] created a facile designed 3D substrate for SERS detection of beta-carotene. The SERS devices had high-density, uniformly distributed hotspots of gold (Au) multibranches, which were electroless plated with a nanoporous polymer as a template. The sensitivity, stability and reproducibility were investigated. The new approach provided new substrate fabricating method for the analysis of analytes with an enhancement factor of 10^8^.

SERS was also used to detect butylated hydroxyanisole (BHA), generally used in foods or oil products to avoid oxidation [98]. Direct determination of BHA in several edible and essential oils without any sample treatment was realized by using SERS with a low-cost homemade silver substrate. The detailed conditions of experiment, characterics of every kind of oil, and the traits of substrate were showed and discussed [99]. Interaction of targets and nanomaterials is crucial for the improvement of the LOD of a SERS based detection. Studies on SERS detection of catechin showed that the ratio of catechin to AgNPs and NaCl was important for the data quality. Raman signal was detectable at 10^−18^ M level at the optimized ratio. Raman detection of catechin with high sensitivity and reproducibility was achieved due to the obvious enhancement of Raman signal [100]. Aguilar-Hernandez et al. [88] systematically evaluated SERS measurements of several examples of antioxidants compounds derived from various plants.

### 3.2. Anthocyanins

Anthocyanins are important natural products leading to the various colors in different parts of plants, including fruits, flowers, and grains [101,102]. Anthocyanins are derivatives of the salts of 2-phenylbenzopyrylium, naturally present as glycosylated molecules [103,104]. Subtle changes of the molecule structure can be revealed in the SERS spectra [105], which demonstrated the advantages of SERS used to identificate anthocyanidins. Benzopyrylium is the common moiety of the molecule structure of anthocyanidins with different phenyl rings, and different kinds of anthocyanidins exist in aqueous extracting solutions with different pH. Compared to resonance Raman, which could not distinguish the similar species, SERS can be used to further identify anthocyanidins. Zaffino et al. [106] identified some kinds of anthocyanidins using SERS and studied the influence of the pH on the SERS spectrum of six main anthocyanidins. They further provided SERS procedures of identificating anthocyanin in plant extracts. Optimized procedures of sample extraction and preparation were selected for different plant species and then detected by SERS measurement [107]. Luca et al. [108] reported the SERS spectra of analytes extracted from mulberry, gromwell, rhubarb and so on. It was proposed that the SERS spectrum was correlated with the molecule structure.

### 3.3. Chinese Herbal Medicinal Ingredients

**Figure 4:**
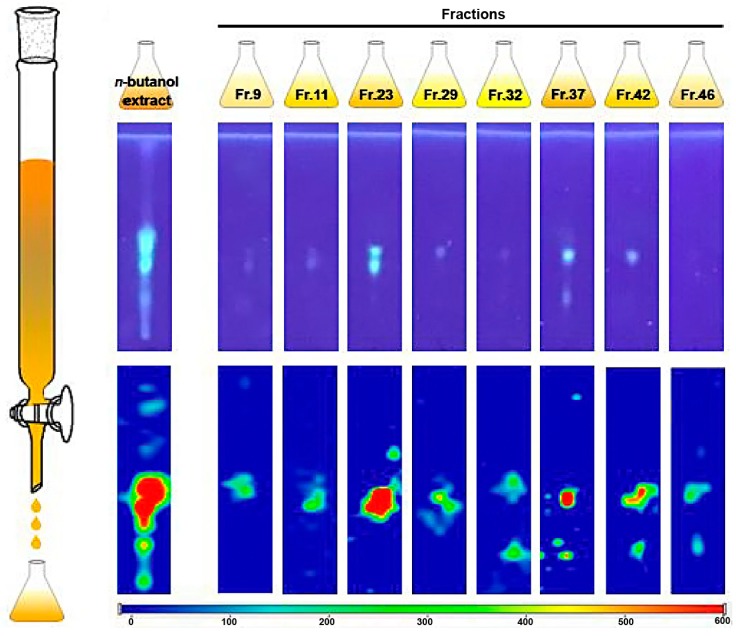
Schematic of the proposed TLC-SERS method for n-butanol extract detection [109]. Reproduced with permission from [109]. Copyright Elsevier, 2018.

SERS is also used to characterize herb extracts. Gu et al. [109] presented an analytical method for the rapid detection and identification of bioactive substances from Chinese herbs by combining thin layer chromatography (TLC) and SERS. The limits of detection were 0.05–0.10 μM, which were far more sensitive than the UV lamp based method. The established method further enabled predicting and uncovering of unknown substances from Chinese herbs. The TLC-SERS strategy for the sample preparation procedure of *n*-butanol was illustrated in Figure 4.

Zhang et al. [110] employed SERS and fluorescence spectroscopy to test the interaction of herb molecules with human serum albumin (HSA). The SERS methods were applied to predicting the molecule conformation on colloidal AuNPs. Similar transformations were found for four ginsenosides when combined with HSA, while the glucose and aglycone were exposed to fit suitable sites. Zheng et al. [111] developed a novel SERS method to monitor 5-demethylnobiletin produced in citrus and the SERS methods based on substrate or solution were all well correlated with high performance liquid chromatography (HPLC). The solution-based SERS method separated nobiletin by applying a procedure like “affinity chromatography”. The substrate-based SERS could simply and quickly collect the “fingerprint” spectra. SERS had more advantages than HPLC methods in convenient, rapid characterizition and quantification of the production of 5-demethylnobiletin.

### 3.4. Molecular Fingerprint Identification of Plant

The fingerprinting method is widely applied to the determination of molecules or the bond behavior in samples. A new SERS method was proposed to get the spectrum of tea species to detect the sample purity involved in different types of planting and processing. The fingerprint SERS spectrum of seven kinds of tea samples was obtained. Data processing method of Principal Component Analysis (PCA) was used to separate tea species and several models for different tea samples. The combined method of fingerprinting-PCA was accurate and rapid for the evaluation of different tea species [112,113].

Pollen extracts from various plant species have different beneficial biological effects [114]. Seifert et al. [115,116] analyzed aqueous extracts from different kinds of pollen using SERS with AuNPs substrate. The SERS spectra of targets were specific, and the different species could be distinguished to classify the genus. The accurate identification of pollen was achieved by analyzing the intrinsic information in SERS data. Results showed that SERS had good potential to characterize and identify pollen species, and could improve the study of pollen physiology. Routine investigations of food composition and vitamin/nutrient contents are challenged by food matrix complexity and low analyte content in samples. Radu et al. [117] simultaneously detected two B-vitamins using the SERS fingerprint method, which could sensitively identify analyte molecules at a low cost.

### 3.5. Other Plant Components

SERS spectra of DNA can offer an assessment of the genetic identity of different kinds of plants [118,119]. Knowledge of genetic resources with high diversity is a precondition for developing novel species. Muntean et al. measured the half bandwidths of SERS of genomic DNAs in vitro-grown tissues of apple leaf. Results showed that the SERS method could be applied to studying the dynamics of DNA approaching the surface of the metallic substrate, with good perspectives of analyzing interactions of DNA-ligand or changes of DNA structure under environmental stress conditions [120,121]. They extended the application of this SERS method to other plant leaves, such as chrysanthemum, common sundew, edelweiss and so on [122]. The SERS spectra of genomic DNAs from tomato plants was also collected and the structural changes of DNAs undergoing cryopreservation were discussed [123]. Based on these works, they put forward that interactions of plant DNA and ligand or the precision DNA structure when approaching metallic surface could be further studied using SERS.

SERS can be used to investigate samples with weak Raman signals, for example juices and pulp. Camerlingo et al. [124] studied apple juices and pulp to verify the existence of fructose and pectin, which were related to the quality evaluation of these products. A home-made substrate fabricated with a glass slide decorated with AuNPs was designed and applied to the SERS detection. The obtained SERS spectra with legible Raman features provided useful information for the characterization of products in food processing. 

Peanuts are a main life-threatening food allergen [125,126]. Gezer et al. [127] applied a biodegradable SERS technique to detecting Ara h1, the main kind of allergen protein, with LOD of 0.14 mg/mL. By using anti-Ara h1 monoclonal antibodies to functionalize the sensor surface, high specificity was achieved.

Palanco et al. [128] used plasmonic structures of silver nanoaggregates or films to enhance the detection of the chemical components of an onion layer. Results showed a competitive adsorption of molecules of onion and reporter. Different spectra from different parts of the layer indicated the complicated molecule structure of the plant. Shen et al. [129] presented nondestructive imaging of the living leaf using micro-Raman spectroscopy by delivering the carbon-encapsulated SERS tags into the living leaf. In vivo SERS spectra were used to investigate the distribution of tags, which could avoid interfering from autofluorescence. The novel modality of imaging provided SERS attractive potential for noninvasive biochemical imaging of living plants.

Cepeda-Perez et al. [130] reported the distribution and interaction of quantum dots (QDs) in the microalgae extracellular matrix. Changes in the Raman spectra of *Haematococcus pluvialis* microalgae caused by the adsorption of QDs were found by applying nano-sensors with bare anisotropic gold structures for SERS effect. This research demonstrated early QDs accumulation in plant cells which would benefit understanding of the environmental influence [131].

## 4. Application on the Detection of Microorganism Original Biomolecules

### 4.1. Bacteria Original Biomolecules

**Figure 5:**
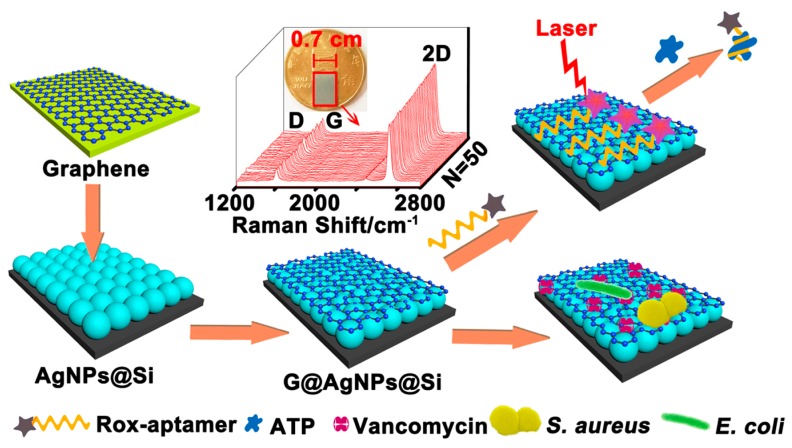
Schematic of the chip system for the analysis of ATP and bacteria [133]. Reproduced with permission from [133]. American Chemical Society, 2018.

Sensitive and accurate pathogen detection is a key measure for ensuring public health due to the rapidly increasing infectious disease rate globally. The correct identification of pathogens in clinical or food samples assures the proper selection of clinical treatments or food safety procedures [132]. SERS is well-suited for bacteria detection from molecular to cellular level due to its sensitivity, selectivity, and compatiblity with other techniques. A representative work was presented by Meng et al. [133] in which a new type of SERS chip, consisting a sophisticated sandwich graphene (G)-AgNP-silicon (Si) nanohybrids, has been developed. The chip system could achieve both molecular detection and cellular analysis in samples. A schematic illustration of the developed platform was showed in Figure 5. The chip could realize sensitive and accurate quantification of adenosine triphosphate (ATP), with LOD of about 1 pM, and can also simultaneously capture, discriminate, and inactivate the bacteria. The efficiency of bacteria capture was 54% at the bacteria concentration of 10^8^ CFU/mL, and 93% antibacterial rate could be reached 24 h after treating.

For the application of SERS in the bacteria sensing, most of the studies focus on the cell detection, which is through the recognition of biomolecule on the cell membrane or inside the cell using antibodies, molecularly imprinted polymers or aptamers. The bacteria SERS signal can be significantly enhanced by these specifically recognized molecules, and the bacteria could be identified directly and visually from the SERS spectra [134]. There have been several reviews about the bacteria cell detection using SERS. Efrima et al. [135] reviewed studies using SERS for bacteria identification based on analyzing the spectra according to the nature of active centers and their distribution in the bacterium. Chauvet et al. [136] reviewed and proposed main strategies, such as methods to prepare the sample, from the bacterial culture conditions to the analysis of the spectra over the last 20 years. These reviews introduced the most recent reports about SERS detection of bacteria cells, which were comprehensive and detailed. So here the application of SERS on the detection of bacteria original biomolecules, rather than cells, was reviewed. 

#### 4.1.1. Bacteria DNAs

Enteritidis caused by *Salmonella enterica* is a common foodborne disease increasingly rising globally [137]. Draz et al. [138] established an integrated assay for DNA detection of *Salmonella*. Using Au-nanoprobes, the LOD of the developed method (66 CFU/mL) was about 100-fold lower than the conventional PCR method.

Gracie et al. reported a novel detection method of three meningitis pathogens using lambda-Exonuclease digestion of double-stranded DNA and SERS detection. Two complementary DNA probes were simultaneously hybridized to a target sequence. The obtained double stranded DNA was digested using lambda-exonuclease and then detected by SERS. Meningitis pathogens were detected with LOD in the range of pico-molar [139].

Another simple and low-cost platform was designed to sensitively detect bacterial DNA by SERS using AuNPs modified as reporter probes, by using the separation and enrichment function of magnetic beads. A good linear relationship was gained of the DNA concentration range of 5 pM to 5 nM. The LOD for the detection of bacterial DNA was about 5 pM [140].

#### 4.1.2. Bacteria Proteins

Wang et al. [141] adopted a newly produced nanoyeast single-chain variable fragment to replace antibody, which is specific, cost-effective, and stable. By combining SERS with a microfluidic chip using nanoparticle clusters as labels, a universal platform for the sensitive and specific detection of pathogen antigens was established. LODs were 1 pg/mL for *Entamoeba histolytica* antigen EHI 115350 and 10 pg/mL for EHI 182030.

Trypsin shaving, as a targeted proteomic method, can be used to identify bacterial proteins exposed on cell-surface. For the redox-active proteins, obtained datasets were matched with SERS to identify the cofactors relevant with the cell-surface proteins. Further, this method could help solve problems concerning the existence of electron transport molecules in bacteria, especially microorganism that oxidize metals or metalloids [142].

#### 4.1.3. Other Bacteria Component Molecules

The bacterial outer membrane is composed of biochemical compounds that have specific information about bacterial strains, different stages of growth, responses to stimulation and so on [143]. Xu et al. [144] proposed that the molecule information of the bacterial outer membrane could be applied in rapidly detecting and identifying bacteria using the SERS method. Seven strains of the marine pathogen were used as models. Based on the SERS spectra, barcodes were generated for the detection of individual bacterial strains in blind samples. The developed sensing methods had broad applications in the areas of biomedical diagnostics, environmental monitoring, and security aspects. Just as proposed in the above report, SERS is a rapid and sensitive method with the potential to detect chemical changes on the surface of bacterial cell induced by the environmental changes. Stephe et al. [145] classified fourteen Arthrobacter strains with up to 97% accuracy by adopting PCA in combination with Linear Discriminant Analysis. Results showed that SERS could be used as a valuable tool to monitor and characterize phenotypic variations when faced with different environmental circumstances. A lab-on-a-chip (LOC)-SERS device was developed to differentiate six species of *mycobacteria*. The easy and reliable system was fabricated by the combination of a bead-beating module for cell disruption with the LOC-SERS device. Without analyte extraction or other treatments, the SERS spectra can quantify mycolic acid as the cell-wall component. By recording a data set using the LOC-SERS device, the type differentiation could also be achieved. At least 2100 SERS spectra could be obtained in 1 h [146]. Moreover, a reconfigurable assay was proposed to identify and monitor bacteria by direct detection of bacterial volatile organic compounds via SERS. Highly clinically relevant organisms were used to distinguish the species of bacteria with LOD of *Escherichia coli* at 10 CFU/mL in 12 h [147].

#### 4.1.4. Bacteria Metabolites

Determination of the chemical composition of biofilm matrices is crucial in different biological fields. The information of biofilm development and composition will help to select proper eliminating measures. Quorum sensing is significant in the survival of bacteria in biofilms and can be revealed by detecting related signaling metabolites. Multifunctional platforms for real-time tracing of metabolites secretion in biofilms were desired. Guo et al. [148] made a flexible and sticky sandwich note with two pieces of hexagonal boron nitride layers packaging gold nanostars, which can stick on natural biofilms for metabolites monitoring by SERS imaging sensitively. The sticky note can accurately quantify *Pseudomonas aeruginosa* after 1 h growth of biofilm by using its pyocyanin secretion as an internal standard for SERS signal self-calibration. This universal SERS sticky note can be used as a versatile tool in bacterial behavior research. 

The metabolites of purine degradation excited at 785 nm are the major molecular species dominating the SERS spectra of bacteria. These molecules are produced by the bacterial starvation response in pure water washes following enrichment in culture mediums. The enzymes of bacterial supernatant that plays a main role in the known purine metabolism pathways were detected by SERS spectra to determine the bacterial specificity. These results showed that SERS could be a rapid diagnostic tool for metabolic profiling [149].

Zukovskaja et al. [150] developed a microfluidic device combined with SERS to analyze a *Pseudomonas aeruginosa* specific metabolite in aqueous solution with a LOD of 0.5 µM [151,152]. A simple and novel SERS platform is fabricated for in situ monitoring the nitric oxide (NO) release of a single bacterium. NO released under antibiotics and co-infected bacteria stress was investigated using this method [153]. *Staphylococcal* Enterotoxin B (SEB) was detected ultrasensitively using the SRES sandwich immunoassay. The antibody and SERS reporter molecule modified magnetic gold nanorod particles were used to capture SEB. A good linear relativity between the SEB concentration and SERS signal was found and the LOD was 768 mM [154].

For the SERS detection of different types of bacterial component molecules, more sophisticated nanomaterials, complicated microfluidic chips [133], and new types of recognition molecules such as nanoyeast single-chain variable fragment [141], were designed, fabricated, and used to prepare the SERS-active substrate.

### 4.2. Virus Original Biomolecules

Virus infection leads to severe epidemics of a large number of worldwide populations each year with high morbidity and mortality. Sensitive and simple detection of viruses is vital for control of viral spread at an early stage [155]. Virus detection methods are usually based on the immunoassay and PCR [156,157]. Considering the fact that they are rapid, portable and sensitive, the SERS-based immunoassay or PCR methods have potential application as a POC test in diagnosis.

#### 4.2.1. Virus Proteins

**Figure 6:**
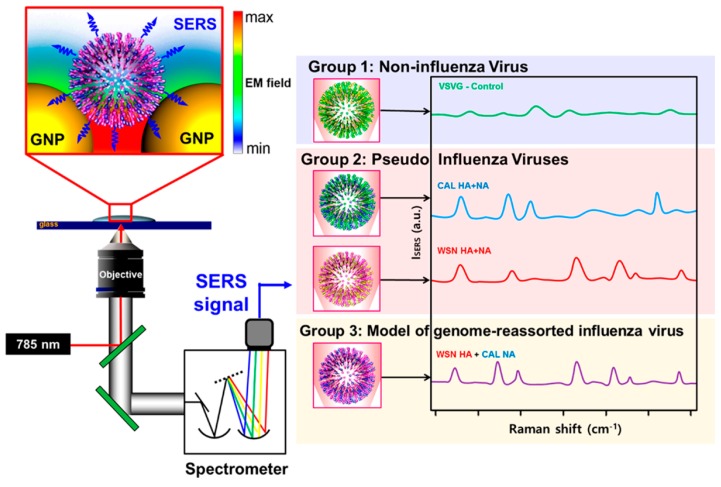
Schematic of the rapid detection of influenza viruses via SERS [160]. Reproduced with permission from [160]. American Chemical Society, 2015.

Many kinds of virus surface proteins, such as G protein and antigen, were used to identify virus [158,159]. Lim et al. [160] tested the hypothesis that surface proteins and lipids of newly presented influenza viruses could enhance Raman peaks on AuNPs, which could then be distinguished from those of pseudotype with a noninfluenza virus component (Figure 6). This work provided a powerful label-free SERS platform to rapidly identify emerging influenza viruses. 

A sensitive immunoassay with SERS detection combined with microfluidic devices was developed using a novel Raman reporter molecule and substrate. Basic fuchsin (FC) was designed as Raman reporter, which can notably enhance SERS signal and connect the antibody and gold nanostructures. A good linear relativity between the intensity of SERS signal of FC band and concentration of Hepatitis B virus antigen was obtained with LOD of 0.01 IU/mL [161].

Sun et al. developed a magnetic immunosensor with SERS detection of intact and inactivated influenza virus H3N2 by fabricating a sandwich complex combining SERS tags, target viruses and supporting substrates. Using a portable Raman spectrometer, a good linear relationship was obtained with a LOD of 10^2^ TCID50/mL [162].

Porcine circovirus is a ubiquitous and crucial infectious virus in global pig farms [163,164]. Luo et al. reported an immunoassay combined with SERS detection for porcine circovirus type 2 (PCV2) using multi-branched mb-AuNPs combined with the PCV2 cap protein antibody as substrates and Raman reporters. A calibration curve plotting the intensity Raman signal at 1076 cm^−1^ versus the concentrations of PCV2 was obtained from 8 × 10^2^ to 8 × 10^6^ copy/mL with the LOD of 8 × 10^2^ copy/mL. This method is rapid, facile and sensitive compared to conventional PCR method [165]. They further used porous carbon films coated with AgNPs to determine PCV2, porcine parvovirus (PPV) and porcine pseudorabies virus (PRV). The LOD was improved as low as 1 × 10^7^ copy/mL [166]. Enterovirus 71 (EV71), another health hazard, needs to be monitored with rapid POC detection. Reyes et al. developed a SERS method utilizing colloidal gold nanostars (AuNS) aggregation induced by protein for rapid detection of EV71 without substrates, Raman labels or sample preparing. AuNS was modified with scavenger receptor class B, member 2 (SCARB2) protein. In the absence of EV71, AuNS modified with scavenger receptor class B member 2 (SCARB2) protein aggregated produced four enhanced Raman peaks at 390, 510, 670, and 910 cm^−1^. In the presence of EV71, as the virus bound to AuNS-SCARB2 preventing aggregation, the peak at 390 cm^−1^ diminished in intensity with other three peaks disappeared, which could be potential indicators for the specific detection of EV71. EV71 could be detected in protein-rich medium within 15 min with this facile approach [167]. Sanchez-Purra et al. [168] explored the high sensitivity of SERS in a universal approach that could distinguish Zika from dengue nonstructural protein 1 (NS1) biomarkers. SERS-encoded gold nanostars were modified with the antibodies of both viruses for a dipstick immunoassay with 15-fold and 7-fold lower LOD for Zika NS1 and dengue NS1, respectively.

#### 4.2.2. Virus DNAs

With the distinguished spectral properties of metal carbonyls, Lin et al. [169] proposed a SERS ratiometric assay to detect cell-free circulating DNA (cfDNA) derived from the Epstein-Barr virus in human blood samples for nasopharyngeal cancer. Rhenium carbonyl (Re-CO) was used as a DNA probe, and osmium carbonyl (Os-CO) was used as an internal reference. The binding of Re-CO to cfDNA is accompanied with a performance of a stretching vibrations peak at 2113 cm^−1^ overlapping with Os-CO (2025 cm^−1^). This led to an increase in the ratio of I-2113/I-2025, quantitatively corresponding to the increase of cfDNA. The SERS method can be applied to detecting cfDNA in clinical blood samples because the ratio of I-2113/I-2025 lying in the region of 1780–2200 cm^−1^ of the biomolecules.

An indirect capture method was established using colloidal AuNPs for SERS detection of DNA. The capture sequence obtained from the RNA genome of West Nile Virus. Colloidal gold was modified with a complementary capture oligonucleotide and a reporter oligonucleotide combined with methylene blue as Raman label. The LOD was in the submicromolar range [170].

To obtain sensitive, stable and reproducible gene detection of respiratory syncytial virus (RSV), a new SERS substrates were fabricated by electroless metallization of Ag and vapor phase deposition of Au on the nanostructured templates. Gene detection of RSV was achieved with a molecular probe with a fluorescent moiety and a linker to be attached on the substrate. To detect multiple targets, molecular probes were designed using a broad range of fluorophores. This reproducible dual-mode method (i.e., fluorescent and SERS) was self-confirmatory and can eliminate false positives [171]. 

For the SERS detection of virus biomolecules, the newly developed free-substrate method based on the AuNS aggregation induced by protein was used to achieve the rapid detection of EV71 [167]. Label-free SERS platform, microfluidic chip with new structure and new type of DNA probe were also applied to detect virus biomolecules.

### 4.3. Other Microorganism Original Biomolecules

Fungal infections cause high morbidity and mortality among hospitalized patients and immunocompromised individuals; fast and accurate diagnosis for fungal diseases is in demand [172]. SERS combined with PCA was used to detect and identify human fungal pathogens rapidly and reliably. Dina et al. [173] distinguished different clinical samples of fungal species using the chemometrics assisted SERS-based method. The overall analysis of the SERS spectra was carried out using appropriate chemometric tools-classical and fuzzy PCA combined with linear discriminant analysis to analysis the first principal components. Discrimination between several species of fungal pathogen strains showed that the established method could be applied as an alternative routine analysis tool in clinical diagnosis. Witkowska et al. [174] confirmed that the SERS method could effectively distinguish between certain fungal pathogens and offer taxonomic relation of fungi. Moreover, using the PCA analysis, statistical classification of fungi could be performed. Two principal components calculated were the most clinically significant, displaying 97% of the variability and discriminate between fungal species.

Zivanovic et al. [175] reported the molecular composition probing of Leishmania-infected macrophage cells by SERS. The data was used to assess the distribution of cholesterol and ergosterolin the amastigote period of the parasite and its vacuole ambient enviroment. Parasite original proteophosphoglycans, an important infection marker, were identified. Mycoplasma pneumoniae, as a respiratory related pathogen, can cause chronic bronchitis and pneumonia. The main surface protein P1 needed to form complexes with certain proteins to act in receptor binding or motility, and the variability in the related proteins can be used to distinguish the major genotypes. Strains with different genotype can be discriminated sensitively and specifically by using SERS on silver nanorod arrays. Krause et al. [176] applied the variable selection method to the identification of Raman bands vital in the classification of *M. pneumoniae* strains. The current methods for malaria diagnosis are time consuming, and are not suitable for early disease diagnosis. Garrett et al. [177] developed a reliable method based on a novel gold-coated SERS substrate and applied to the detection of malarial hemozoin pigment in the blood samples with 0.005% and 0.0005% infected cells.

## 5. Conclusions and Outlook

The recent explosive growth of the SERS studies brings these sensitive, rapid, accurate and reliable methods to more application fields. The merits of SERS expand its potential in the identification and quantification of various Raman-active biomolecules. Based on the development of new SERS strategies and combined with other techniques (e.g., immunoassay, PCR, and microfluidic chip), SERS shows increasingly broad application in detecting biomolecules from humans, animals, plants, and microorganisms, which can contribute to improving food safety, clinical diagnosis, and environmental monitoring. To be specific, SERS spectrum is able to provide abundant clues about the structure and/or quantity of interested molecules, including the hazard(s) or nutrient(s) present in foods, the pollutants in environmental samples, pathogens in clinical samples or biological processes occurring at the cellular or molecular level, etc. 

This review article provided an overview of the recent progress and current shortages of SERS on biomolecule detection in order to indicate the application direction in the future. For example, there is a lack of and an urgent need for SERS application in the basic study of life sciences, such as animal or plant physiology that is crucial for getting more deep and basic knowledge on food security, environmental aspects and the ecological system. Therefore, the target biomolecules in life sciences were selected in this review to attract the attention of experts in both SERS and life science research and to expand the depth and fields of SERS application. There are still some technological problems that urgently require to be solved. Robust and reproducible SERS substrates as the critical parts of SERS methods are still under development, and the property needs to be improved and adapted to a wider range of analytes and biological samples. New types of nanomaterials that can improve the sensitivity, specificity and stability of SERS detection will benefit from the progress in nanotechnology and nanofabrication. More and more sophisticated nanostructures will be successfully fabricated and the problems of inconsistency and production cost of using SERS-active substrates will be gradually solved. The application of SERS in the detection of biology molecules was also seriously limited and challenged by the complex matrix interference of biology samples. The development of more effective sample preparation and purification methods that can be integrated with SERS detection would allow for a wider application range of SERS detection of biological molecules. To achieve the efficient extraction and purification of target analytes, the selection of specific biological or biomimetic recognition molecules, such as antibody, molecularly imprinted polymers, and aptamer with strong affinity and specificity, and their application on the modification of SERS substrates will be important technological factors. Furthermore, through the use of flexible substrates (e.g.*,* paper and plastic films), sensitive, low cost and disposable commercial SERS platforms will be developed to adapt to POC applications in resource-limited settings. Finally, combined with the development of nanotechnology and biochemical methodology, SERS holds great promise in showing superior capabilities in biological fields in future.
